# ARIMA MODEL IN PREDICTING OF COVID-19 EPIDEMIC FOR THE SOUTHERN AFRICA REGION

**DOI:** 10.21010/Ajidv17i1.1

**Published:** 2022-12-22

**Authors:** SHOKO Claris, NJUHO Peter

**Affiliations:** Statistics, University of South Africa, Pretoria, South Africa; Statistics, University of South Africa, Pretoria, South Africa

**Keywords:** ARIMA, Box-Jenkins, Forecast, SADC

## Abstract

**Background:**

Coronavirus pandemic, a serious global public health threat, affects the Southern African countries more than any other country on the continent. The region has become the epicenter of the coronavirus with South Africa accounting for the most cases. To cap the deadly effect caused by the pandemic, we apply a statistical modelling approach to investigate and predict COVID-19 incidence.

**Methods:**

Using secondary data on the daily confirmed COVID-19 cases per million for Southern Africa Development Community (SADC) member states from March 5, 2020, to July 15, 2021, we model and forecast the spread of coronavirus in the region. We select the best ARIMA model based on the log-likelihood, AIC, and BIC of the fitted models.

**Results:**

The ARIMA (11,1,11) model for the complete data set was finally selected among ARIMA models based upon the parameter test and the Box–Ljung test. The ARIMA(11,1,9) was the best candidate for the training set. A 15-day forecast was also made from the model, which shows a perfect fit with the testing set.

**Conclusion:**

The number of new COVID-19 cases per million for the SADC shows a downward trend, but the trend is characterized by peaks from time to time. Tightening up of the preventive measures continuously needs to be adapted in order to eradicate the coronavirus epidemic from the population.

## Introduction

Sixteen countries constitute the Southern Africa Development Community (SADC) Member States; namely Angola, Botswana, Eswatini, Comoros, Democratic Republic of Congo (DRC), Lesotho, Madagascar, Malawi, Mauritius, Mozambique, Namibia, Seychelles, South Africa, Tanzania, Zambia, and Zimbabwe. The first case of COVID-19 in the SADC region was recorded in early March 2020 in South Africa and the numbers have been increasing exponentially (WHO, 2020). With the exception of Comoro and Lesotho, other member states had been affected by the epidemic by the 15^th^ of April 2020 (WHO, 2020).

The coronavirus pandemic has resulted in complex challenges across the world, and the SADC region has not been spared. Various measures have been undertaken by the SADC Member states and these include preparedness and response mechanisms, awareness programs, suspension of inbound and outbound flights, suspension of business and tourism travel, set up of border and in-country testing centres, social distancing, and cancellation of gatherings, adoption of self-isolation and mandatory quarantine for a minimum of fourteen days, and treatment for those that test positive (WHO, 2020).

The Southern Africa region has been hit hardest by the COVID-19 pandemic in Africa, thus the epicenter of the coronavirus in the African continent (Massinga *et al*. 2020). By February 2021 the SADC region had accounted for half of the reported cases in Africa. Of the five African countries accounting for close to 76% of new infections, three of them are members of the SADC namely South Africa, Zambia, and Namibia (UN Economic Commission for Africa, 2020).

With COVID-19 becoming one of the most serious global public health threats, investigating and predicting COVID-19 incidence contributes to the control of its spread. Modelling a future forecast that estimates the regular number of confirmed cases enhances the implementation of rules aimed at controlling the spread of COVID-19. Statistical forecast models play a role in predicting future epidemic threats, managing of societal, economic, cultural, and public health matters. Katoch and Sidhu (2021) predicted the spread and the final size of the COVID-19 epidemic in India using the ARIMA model. Singh *et al*. (2020) predicted the daily confirmed COVID-19 cases for Malaysia using the ARIMA model.

In Bangladesh, Kundu *et al*. (2021) forecasted the expected number of total confirmed cases, total confirmed new cases, total deaths, and total new deaths in Bangladesh.

The ARIMA model, generally known as the Box-Jenkins methodology is used for forecasting and analysis in the time series approach. We model the number of daily COVID-19 cases per million in the SADC region using the ARIMA (p,d,q) models. First, we model for all combined SADC countries than for each of the member countries. We finally forecast the spread of the pandemic using data from the three SADC countries (South Africa, Zambia, and Namibia) reporting high cases of the COVID-19 pandemic.

## Materials and Methods

### ARIMA Model

Oftentimes, the ARIMA models are used in the analysis and forecasting of the time series, focusing on the random side of the time series. The acronym ARIMA (p,d,q) consist of three main sections:

1) Autoregressive models, AR (p) which express the present value 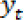 as a linear function in the lagged values of the variable. Thus,


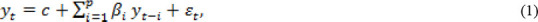


where 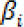 denotes the parameters of the autoregressive, 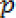 denotes the lag operator, 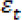 denotes the error terms, and 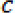 denotes the constant.

2) Integration, I (d) which indicates the degree to which the variable is stationary. Thus,


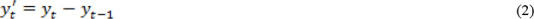


3) Moving Average, MA (q) which express the current value of the variable, 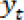 as a linear function in the present value of the random error term and a number of its lag values. Hence,


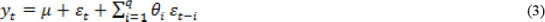


where 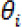 denote the parameters of the moving average and *μ* denotes the expectation of 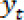 (often assumed to equal 0).

### Ethical Consideration

No formal ethical approval was obtained for this study because all the data used were from a secondary source.

### Estimation of parameters of ARIMA Model

### Process in Stages

The ARIMA models estimation method involves several stages undertaken before making predictions namely the identification, estimation, forecasting, and forecasting validation stages.

(a) *Identification stage* - where the degree of stationary of the variable is determined using Augmented Dickey-Fuller (ADF). Based on the autocorrelation function (ACF) and the partial autocorrelation function (PAC), we first define (d), followed by (p) and then (q).

(b) *Estimation stage –* were using the Maximum likelihood estimation method and based on Akaike information criterion (AIC), the appropriate model is selected after comparing possible models.

(c) *Forecasting stage -* where the prediction is made using the final model.

(d) *Forecasting validation* *stage -* where the model is validated on the prediction based on several indicators that represent the deviation of the calculated values from the actual values, which are the mean absolute error (MAE), root mean square error (RMSE), and mean absolute percentage error (MAPE). The ACF plot ascertains the existence of autocorrelation between the residual values. The validity of the prediction is checked using the plots of the difference between the actual and the forecast. Table 1 presents the definition of indicators used in assessing the validity of the forecasts and associated formulae.

**Table 1:** Definition of indicators, formula and terms used in assessing the validity of forecasts.

Indicators	formula	Define terms
Autocorrelation function (ACF)	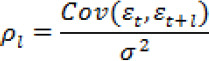	Error terms, 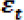 Number of lags, 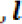
Akaike Information Criterion (AIC)	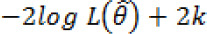	Maximum value of the 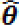 Likelihood function Number of estimated parameters 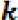
Mean Absolute Error (MAE)	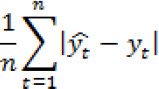	The forecast value 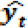 The actual value 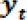 Number of fitted observed ***n***
Root Mean Square Error (RMSE)	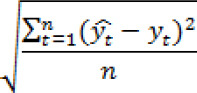	
Mean Absolute Percentages Error (MAPE)	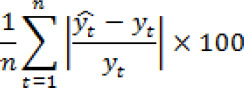	

### Test of stationarity

We test for stationary time series using an Augmented Dickey-Fuller (ADF) test (Dickey and Fuller, 1979). For the transformed nonstationary time series into a stationary time series, we adapt the difference or logarithmic transformation. Achieving stationary is a precondition for establishing an ARIMA model.

### Model identification

Appropriate values of p, d, and q of the ARIMA model were identified as part of model identification. We identified the value of d by the number of differentials, the AR and MA from the autocorrelation function (ACF), and the partial autocorrelation function (PACF) plot against the lag length, respectively. In addition, some model selection criteria such as the Akaike information criterion (AIC), and Bayesian information criteria (BIC) were used. The optimal model was chosen based on the smallest value of AIC and BIC (Akaike, 1974).

After the identification of appropriate values of p and q, the next step was to estimate the parameter of the autoregressive and moving average terms included in the model. This was done using the maximum likelihood estimation method.

### Diagnostic checking

The identification and fitting of the ARIMA model and estimation of the parameters preceded the checking of whether the residual series was a white noise using the Ljung-Box Q test. Failure to reject the null hypothesis of white noise led to accepting the fitted model. The Ljung-Box Q statistics is defined as


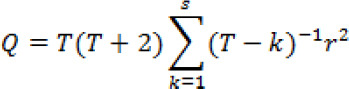


where, *T* is the number of observations, *s* is the length of coefficients to test autocorrelation, *r* is the autocorrelation coefficient for lag k. The Ljung-Box Q statistics follows approximately the chi-square distribution with *(k-q)* degrees of freedom, where *q* is the number of parameters to be estimated (Ljung and Box, 1978).

## Results

### Descriptive statistics

In this study, we used an openly available daily number of confirmed cases of COVID - 19 per million reported by Our World in Data (www.ourworldindata/coronavirus-source-data) from 7 March 2020 to 3 August 2021. Table 1 presents the summary statistics (including the mean and median).

**Figure 1:**
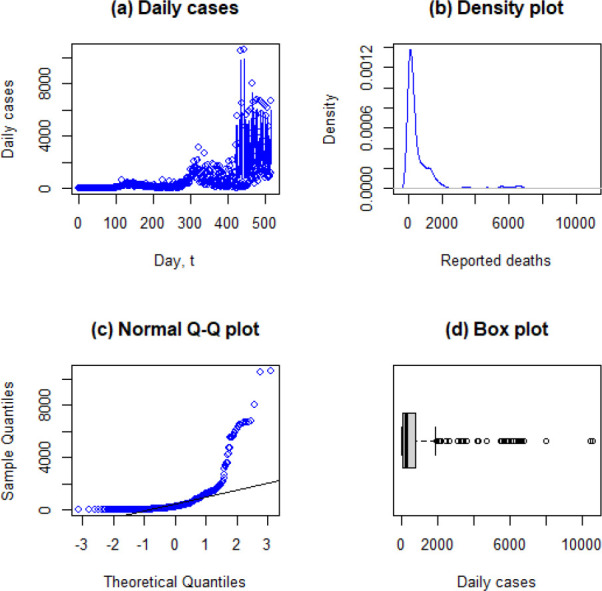
Checking for normality of the time series data.

Results from Table 2 shows that the number of reported daily COVID-19 cases per million in the SADC region ranged from 0 to 18663.85 with an average of 760. 03 per million cases reported each day from the 7^th^ of March, 2020 to the 3^rd^ of August, 2021. Figure 1 presents a plot of the daily cases, the density plot, normal Q-Q plot and boxplot. We use these plots to check for normality of the time series data.

**Table 2:** Descriptive Statistics for COVID-19 per million cases in the SADC region

Minimum	First Quartile	Median	Third Quartile	Maximum	Mean
0	89.73	251.00	803.69	18663.85	760.03

The density plot and the box plot show that the data is skewed to the right and therefore not normality distributed. The normality Q-Q plot also shows some deviation from normality.

### Modelling new COVID-19 cases per million for the SADC region

We start by testing for the stationarity of the original time series data and also that of the differenced time series data. We do this using the augmented Dickey-Fuller (ADF) test and the Kwiatkowski-Phillips-Schmidt-Shin (KPSS) test at 5% level of significance. Table 2 presents results of the ADF and KPSS tests where ADF test for non-stationary and non-seasonal and KPSS test for stationary and non-seasonal in the time series data.

Table 3 results show that the test for stationarity for the original time series on the number of daily new COVID-19 cases per million for the SADC region confirmed stationarity (Dickey-Fuller = -4.3812, Lag order = 7, p-value = 0.01). However, the KPSS test shows that the original data is non-stationary. The first difference of the data is performed and both the ADF and KPSS test show that the differenced series is stationary in its mean and variance at a 5% level. Therefore we adopt d = 1 for ARIMA (p,d,q) model.

**Table 3:** Test for stationarity of the original time series data and the differenced data.

Tests	Cases per million	Differenced cases per million
ADF	-4.3812 (0.01)**	-10.808 (0.01)**
KPSS	5.0306 (0.01)	0.014221 (0.1)**

We assessed the performance of a number of fitted ARIMA models based on the ME, RMSE, MAE, and MASE. We started off by automatically fitting the ARIMA model for the data. Table 3 presents the result which suggests the candidate ARIMA models.

From Table 4 results, the ARIMA (11,1,11) has the lowest RMSE hence the best candidate to explain the daily COVID-19 cases per million for the SADC region. We further investigate the fitted model by examining the residuals plot and performing the Box-Ljung test. Figure 3 presents residual diagnosis results for the ARIMA (11,1,11) model.

**Table 4:** Selection of the candidate ARIMA (p,d,q) model.

Model	ME	RMSE	MAE	MASE	ACF1
Auto ARIMA(4,1,4)	69.0079	1101.021	435.1874	0.551116	0.02744
ARIMA(9,1,9)	67.80244	1020.430	409.638	0.518761	-0.0032
ARIMA(11,1,11)	62.25194	1005.022	393.7258	0.49861	-0.00592

**Figure 2:**
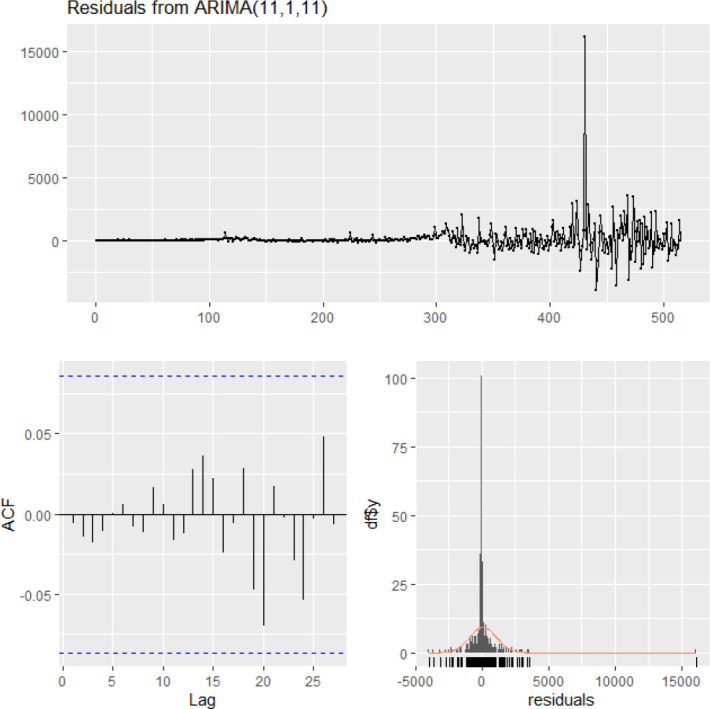
Residual diagnosis for the ARIMA(11,1,11) model.

From Figure 2, the ACF plot of the residuals from the ARIMA(11,1,11) model shows all autocorrelations to fall within the threshold limits, an indication that the residuals are white noise. The Box-Ljung test returns a large p-value 
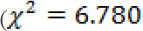
 *df = 20, p-value = 0.9973*), also suggesting that the residuals are white noise. We opt to use a training and a test set rather than time series cross-validation because the series is long.

### Training and testing sets

Using the ARIMA models fitted for the cross-validation time series, we set the criteria for the training data at the first 90% (observations 1 to 498) and for the test data at the last 10% (observations 499 to 515). Table 4 present results from the fitted models ARIMA(9,1,9), ARIMA(11,1,9) and ARIMA(11,1,11), using the training data. The corresponding ME, RMSE, MAE, MASE, and ACF1 for each of the fitted models are included.

Results in Table 5 show that the three models meet the requirements of white noise for the residual time series (*p-value* > 0.05), thus the RMSE values were compared. An ARIMA(11,1,9) has the lowest RMSE, and hence the best candidate for the forecasting. The model has high accuracy and outperforms the forecasting accuracy of the other two models.

**Table 5:** The candidate ARIMA models fitted using the training data and their corresponding assessment errors.

Candidate ARIMA models	ME	RMSE	MAE	MASE	ACF1
ARIMA(9,1,9)	66.078	1004.582	385.540	0.5374	-0.00213
ARIMA(11,1,9)	67.797	990.479	406.899	0.567	-0.00485
ARIMA(11,1,11))	62.252	1005.022	393.726	0.499	-0.00592

Table 6 presents the coefficients (estimate, standard error, z-value and p-value) for the ARIMA (11,1,9) model for *p=1* to *11* and *q=1* to *9*, fitted using the training data. We test the accuracy of the estimated parameters at 5% significance level.

**Table 6:** Estimated parameters and test statistics for the ARIMA (11,1,9) model fitted using the training data.

AR(p) and MA(q) Models	Estimate	Std. Error	z value	Pr(>|z|)
AR(1)	-0.1755	0.21019	-0.8351	0.403666
AR(2)	-0.1417	0.14252	-0.9941	0.320163
AR(3)	-0.3442	0.13824	-2.4897	0.0127865*
AR(4)	-0.3585	0.05584	-6.4207	1.36E-10***
AR(5)	-0.7355	0.09814	-7.4937	6.69E-14***
AR(6)	-0.5427	0.17467	-3.1073	0.001888**
AR(7)	0.1981	0.14059	1.4087	0.158917
AR(8)	-0.4069	0.17538	-2.3203	0.020325*
AR(9)	-0.2583	0.05284	-4.8892	1.01E-06***
AR(10)	-0.1931	0.09173	-2.1051	0.035283*
AR(11)	0.0702	0.08440	0.8321	0.405334
MA(1)	-0.9170	0.20841	-4.4000	1.08E-05***
MA(2)	0.0252	0.25876	0.0973	0.922452
MA(3)	0.6393	0.12613	5.0680	4.02E-07***
MA(4)	-0.5061	0.21405	-2.3644	0.01806*
MA(5)	0.5584	0.09537	5.8550	4.77E-09***
MA(6)	-0.1260	0.11157	-1.1293	0.258779
MA(7)	-0.5921	0.08531	-6.9405	3.91E-12***
MA(8)	0.7162	0.20083	3.5664	0.000362***
MA(9)	-0.4938	0.18228	-2.7089	0.00675**

The estimated parameters for the fitted models have a magnitude less than one (Table 6). The majority of the fitted models were significant at a 5 % significance level, except for the AR(1), AR(2), AR(7), AR(11), MA(2), and MA(6). The standard errors for the estimated parameters were relatively small indicating less variation.

### Forecasting using the ARIMA (11, 1, 9) model

We carried out forecasting for all the time series data using ARIMA (11,1,9) model. First, we divided the data into two sets of the year, “1991” to”2014” as the training data and from “7 march 2021” to “16 July 2021” as the testing data. The data from “17 July 2021” to “3 August 2021” were used as an out-sample forecast. The residuals of the chosen models were stationary and white noise. Table 6 presents a 15 days forecasting for the daily COVID-19 per million cases using ARIMA (11,1,9) model. We present the point forecast and the corresponding 80 % and 95% confidence intervals, for the observation numbers 499 to 513.

**Figure 3:**
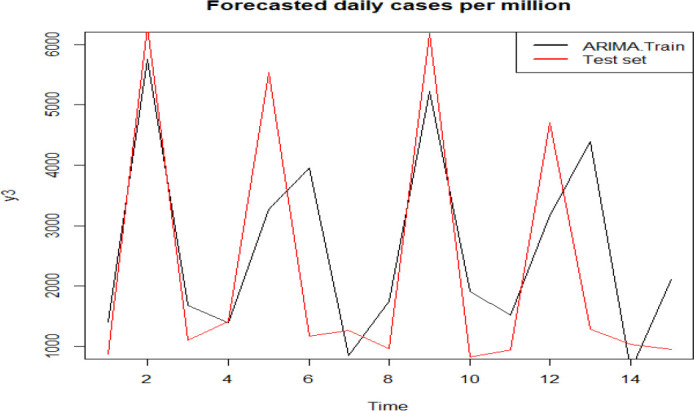
Forecasted daily COVID-19 per million cases for the fitted model from the training set and the test set. Figure 3 shows that all forecasted lines for the training set (ARIMA(11,1,9)) are close to the actual values from the test set, which emphasises the quality of the selected models. The up and down trend characterizes the daily COVID-19 cases per million.

Results in Table 7 show an oscillating trend with the highest peaks expected on the 500^th^ and the 507^th^ days. Smaller peaks occurred on the 511^th^ day of the COVID-19 pandemic. The 95% confidence limit of the predicted values from “7 march 2021” to “16 July 2021”, using the best-fitted models are also presented. Figure 3 presents the predicted values for the forecasted daily COVD-19 per million cases for the fitted model from the training set and the test set.

**Table 7:** 15 day forecasting for the daily COVID-19 per million cases using ARIMA (11,1,9) model.

Observation No.	Point Forecast	80 % [L, U]	95 %[L, U]
499	1404.11	[104.657, 2703.563]	[-583.232, 3391.452]
500	5748.161	[4443.508, 7052.814]	[3752.866, 7743.456]
501	1680.23	[375.414, 2985.047]	[-315.315, 3675.775]
502	1390.971	[-22.331, 2804.273]	[-770.487, 3552.430]
503	3271.865	[1843.662, 4700.069]	[1087.617, 5456.114]
504	3957.906	[2526.972, 5388.841]	[1769.481, 6146.332]
505	851.2315	[-582.394, 2284.857]	[-1341.31, 3043.773]
506	1756.19	[293.728, 3218.653]	[-480.453, 3992.834]
507	5223.569	[3759.226, 6687.912]	[2984.049, 7463.088]
508	1914.037	[441.259, 3386.814]	[-338.382, 4166.455]
509	1517.278	[27.764, 3006.793]	[-760.738, 3795.294]
510	3175.333	[1685.812, 4664.855]	[897.307, 5453.360]
511	4392.675	[2900.543, 5884.807]	[2110.655, 6674.695]
512	615.8379	[-878.250, 2109.925]	[-1669.170, 2900.847]
513	2112.737	[554.377, 3671.096]	[-270.569, 4496.042]

## Discussion

The first COVID-19 case in the SADC region was recorded on the 5^th^ of March 2020 in South Africa. We predict the spread of COVID-19 in the SADC region using reported daily cases per million from the 7^th^ of March 2020 to the 3^rd^ of August 2021. Using time-series data for the 515 observations we provide an appropriate ARIMA model to predict the spread of COVID-19 in the SADC region. Candidate models are designed by observing the spikes from the autocorrelation function (ACF) and partial autocorrelation function (PACF) charts. The Fitting of several ARIMA models resulted in a prediction model whose accuracy in the performance was assessed using RMSEs. We used a training and test set rather than the time series cross-validation because the time series was relatively long.

The use of all data in the analysis provides ARIMA (11,1,11) models that are good for predicting the spread of COVID-19 in the SADC region. Further refinement considering the training set (first 90% of the set) led to an ARIMA (11,1,9) model as the best model. Use of an ARIMA(11,1,9) model in forecasting 15 days in advance fits well with the test (observed) set. An ARIMA (11,1,9) model has a smaller RMSE compared to the ARIMA(11,1,11) model.

## Conclusion

Amongst all the ARIMA models, ARIMA (11,1,9) best predicts the spread of COVID-19 in the SADC region when cases per million are used. A downward trend characterized by peaks from time to time shows the number of new COVID-19 cases per million for the SADC region. The trend suggests a need to continue tightening up the measures that help in mitigating the epidemic. These measures include social distancing, wearing of masks, reducing the number of people in social gatherings, reducing the movement of people between the borders, and so on.

### Conflict of interest

The authors declare that there is no conflict of interest associated with this study.

**List of Abbreviations:**
ACF: Autocorrelation Function;
ADF: Augmented Dickey Fuller
AIC: Akaike Information Criterion;
ARIMA: Autoregressive Integrated Moving Average;
BIC: Bayesian Information Criteria;
COVID-19: Corona Virus Infectious Disease 2019;
DRC: Democratic Republic of Congo;
KPSS: Kwiatkowski-Phillips-Schmidt-Shin;
MAE: Mean Absolute Error;
MAPE: Mean Absolute Percentage Error;
PAC: Partial Autocorrelation Function;
RMSE: Root Mean Square Error;
SADC: Southern African Development Community;
UN: United Nations;
WHO: World Health Organization.
